# Bilateral glomus tumor treated with PET-CT based conformal radiotherapy: a case report

**DOI:** 10.4076/1757-1626-2-8402

**Published:** 2009-09-15

**Authors:** Cem Onal, Oznur Yuksel, Erkan Topkan, Berrin Pehlivan

**Affiliations:** Department of Radiation Oncology, Baskent University Medical Faculty01120, AdanaTurkey

## Abstract

**Introduction:**

Glomus tumors are benign, slow growing tumors originating from paraganglionic tissue, mostly located at the carotid bifurcation, jugular foramen, cervical portion vagus nerve, and middle ear cavity. Radiotherapy is treatment of choice for patients with intracranial extension, and patients with bilateral and multiple tumors, or patients who are inoperable.

**Case presentation:**

We present a 53-year-old female patient with a glomus tumor treated with positron emission tomography computed tomography planning and 3D conformal radiotherapy, and the patient has remained free of disease progression 2 years after.

**Conclusion:**

It is suggested that radiotherapy is a good treatment modality in patients with glomus tumor, and metabolic imaging and treatment planning with positron emission tomography computed tomography is superior to other imaging modalities.

## Introduction

Glomus tumors (GT), also named as paragangliomas and chemodectomas are rare tumors, accounting for 0.03% of all neoplasms and 0.6% of all head and neck tumors [1]. These benign, slow-growing tumors originate from paraganglionic tissue, mostly located at the carotid bifurcation, jugular foramen, cervical portion vagus nerve, and middle ear cavity [2,3]. Typically, these tumors are diagnosed between the fourth and sixth decades of life. Women are affected five to six times more often than men. The most common presentation is painless neck mass.

Familial occurrence is likely to occur in 10% of patients, with an autosomal dominant inheritance. Multiple tumors are seen in 78-87% of familial paragangliomas, and the incidence of bilateral GT is 32% for familial cases and 4% for non-familial cases [4].

To our knowledge, we report the first case of a non-familial bilateral GT treated with Positron Emission Tomography (PET) based 3D conformal radiotherapy (RT), and treatment response assessed with PET.

## Case presentation

**Figure 1.**
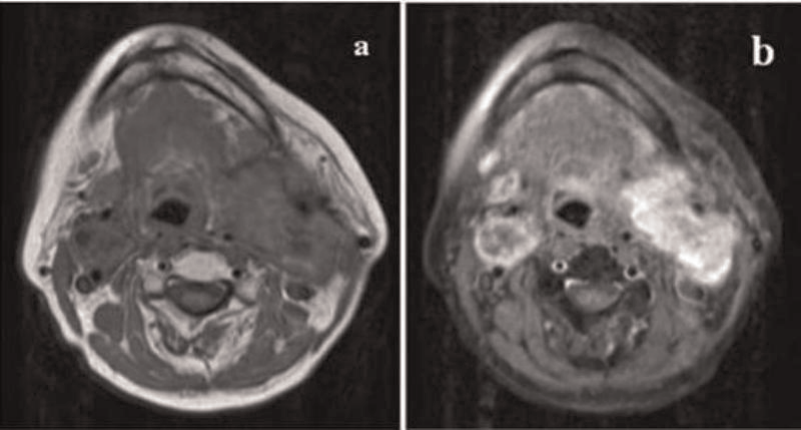
Axial T1-weighted magnetic resonance imaging (MRI) scans. **(a)** A 5 cm hypointense and isointense with muscle mass on the left carotid bifurcation compressing jugulary vein and carotid artery and 2.5 cm mass at the left side. **(b)** These masses intensely enhance contrast.

**Figure 2.**
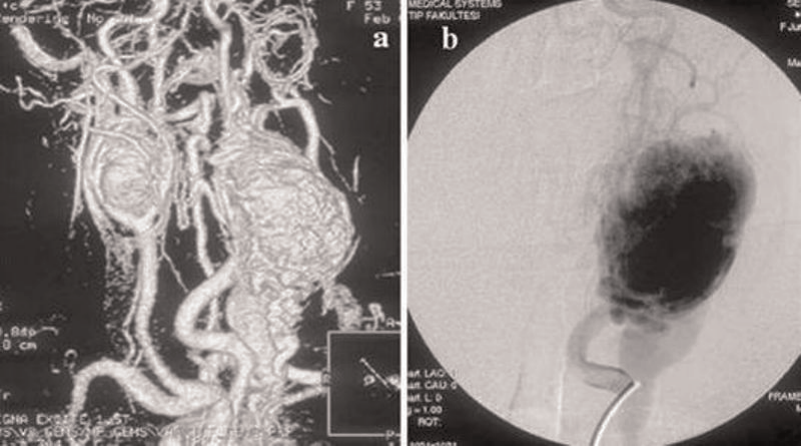
**(a)** Magnetic resonance angiography (MRA) demonstrated a mass at the left carotid bifurcation compressing the external carotid artery, and a mass with similar characteristics at the right carotid bifurcation. **(b)** Lateral angiographic view reveals an intense blush of hypervascular mass.

**Figure 3.**
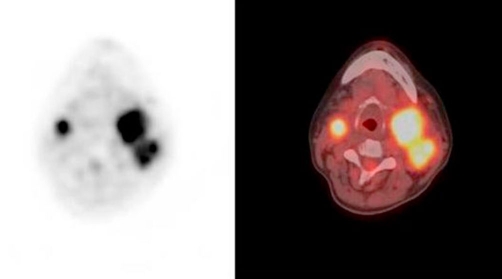
FDG-Positron Emission Tomography (PET) CT scans showed a mass at the left side (SUVmax: 9.9), and mass at the right side of the neck (SUVmax: 8.5).

**Figure 4.**
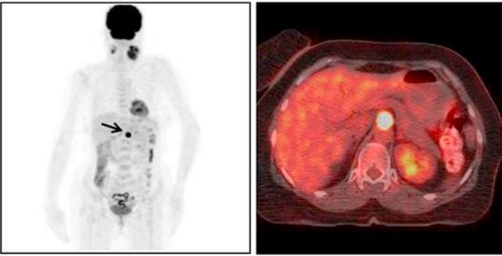
FDG-Positron Emission Tomography (PET) CT scans showed a mass (SUVmax: 28.3) detected at the celiac truncus level in front of the abdominal aorta (arrow).

A 53-year-old Turkish female patient presented with bilateral neck masses for 10 years, which had been increased in size for the last 6 months. Patient was complaining about a moderate hearing loss and tinnitus on the left ear, and pain at the left side of the neck, besides no hoarseness, dysphagia, nasal obstruction and epistaxis were reported. There are no symptoms related with the carotid artery compression. No family history of glomus tumors or any cancers was present. On physical examination, semi-mobile, painless masses with a size of approximately 4 × 3 cm on the right side of the neck, and 8 × 6 cm mass extending from tip of mastoid to level IV inferiorly on the left side were palpated. On systemic evaluation only hypertension diagnosed for 3 years which was under control with medications. 24-hour vanilmandelic acid (VMA) levels and other endocrinologic findings were found to be normal. The magnetic resonance (MRI) studies revealed highly vascularised, intensely enhanced, hypointense and isointense with surrounding muscles 5 cm mass on the left carotid bifurcation compressing jugulary vein and carotid artery, and 2.5 cm mass at the left side (Figures 1a,1b). Cervical Magnetic Resonance Angiography (MRA) demonstrated a 4 × 6 cm mass at the left carotid bifurcation level which compresses the external carotid artery, and a mass with similar characteristics at the right carotid bifurcation (Figure 2a). The internal carotid artery flow at both sides was found to be normal (Figure 2b). On 18F-2-deoxy-D-glucose (FDG) PET-CT, a 6 × 4 cm mass extending from submandibular region to para-laryngeal field at the left side (SUVmax: 9.9), and a 3 × 2.3 cm mass at the right side of the neck (SUVmax : 8.5) (Figure 3). Also an additional 15 mm mass (SUVmax: 28.3) at the celiac truncus level in front of the abdominal aorta was identified besides the bilateral cervical masses (Figure 4). Since MRI and PET findings strongly were correlated with glomus tumor, and there was a high risk of bleeding, tissue diagnosis was not obtained.

**Figure 5.**
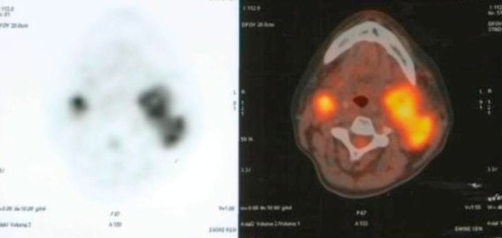
FDG-Positron Emission Tomography (PET) CT scans showed a mass at the left side with necrosis at the central part (SUVmax: 7.7) and a mass (SUVmax: 2.5) at right side of the neck after radiotherapy.

Patient had no gain with medical treatment and embolisation, and the masses were increased in size within last 6 months of period. Since the tumors are located at both sides of the neck, and are close to the vascular structures, the surgeons did not decide to make any surgical procedure; we treated the patient with 3D conformal radiotherapy with a total dose of 50 Gy with 2 Gy fractional daily doses with PET-CT planning. During treatment, acute ‘Radiation Therapy Oncology Group’ (RTOG) Grade II dysphagia and Grade I dry desquamation at neck were seen, which were clarified with adequate medications. At 6th month the pain at the neck region totally disappeared and loss of hearing and the tinnitus at left ear diminished. The PET-CT taken at last visit, revealed a 5 × 4 cm with necrosis at the central part (SUVmax: 7.7) at left side, and a 2 × 2 cm mass (SUVmax: 2.5) at right side of the neck (Figure 5). The patient is still in follow-up and had clinically and radiological stable disease with no complaints after 2 years.

## Discussion

Glomus tumors are mainly presented in two locations; cervical (carotid, vagal) and skull base (jugular and tympanic) [5]. These tumors are rarely seen, and have benign characteristics and a very slow growth rate of 1 mm per year [5]. The initial diagnosis is usually non-tender neck mass, in case of rapid growth or increase in size, because of compression to the surrounding structures, pain, hoarseness, disphagia may develop. Our patient had bilateral neck masses for the last 10 years and a moderate tenderness around the masses because of rapid growth for last 6 months.

CT and MRI are useful diagnostic tools for assessing suspicious paraganglioma. CT can reveal the invasion of the bony structures and intracranial extension, while MRI is better for evaluating vascular structures, extension along neural foramina, and detecting multicentricity [6]. The characteristic appearance of GT in MRI is well-defined hypointense mass with equal signal intensity to adjacent muscle on T1-weighted scans. Intense contrast enhancement is the key finding on MRI. MRA is less invasive diagnostic tool for evaluation of vascular structure of the tumor. With 3D time-of-flight technique, the specificity and sensitivity of MRA was 90% and 92% [7]. Another tool used for diagnosis of GT is PET-CT. Previous reports demonstrated that paragangliomas show uptake of FDG [8,9]. The main advantages of PET-CT are, the metabolic activity of smaller lesions (<1 cm) can be detected with PET-CT, and also with whole body detection it is possible for the proof or exclusion of multifocal or metastatic tumors [10]. In our case, since there was high risk of bleeding, and the patient was inoperable, MRA and FDG-PET were performed for confirmation of diagnosis. Meanwhile, we were suspicious even if there was metastasis or multicentricity, with FDG-PET we detected a nodular lesion at the preaortic region. Also we used PET-CT scans for treatment planning for better target volume delineation.

The optimum management of GT still remains controversial. The treatment options are surgery, RT and watchful waiting. Since the metastasis rate is rare, the main aim of treatment choice is to achieve good local control without increasing morbidity and mortality. Local treatment can be achieved with either surgically as total removal or long-term tumor control and tumor shrinkage with RT. Although good results were achieved with complete surgical removal and total excision with microsurgical approach can often be accomplished, there is a significant risk of morbidity (0-39%) and mortality (0-2.7%) with this approach [11-13]. Radiotherapy is treatment of choice for patients with intracranial extension, and patients with bilateral and multiple tumors, or patients who are inoperable [4,14]. Although the mechanism of growth inhibition of GT is not well understood, vascular elements that comprise the bulk of tumor undergo fibrosis after RT. Furthermore, the tumor (chief) cells are radioresistant and persist after RT [15]. Local tumor control rates were 61-94%, and reported rates of symptom improvement was 71-89% with RT alone [16,17]. It is important to note that the goal of RT is disease control or growth inhibition rather than tumor elimination. Complication rates after RT range from 4% to 10% including dermatitis, mucositis, external otitis and otitis media, altered taste and xerostomia, which often resolve with medication [14,16,17]. Since we made 3D conformal RT, we spare normal tissues better than conventional plan. Only dysphagia and dry desquamation were seen during treatment, which resolved with proper medications.

Glomus tumors respond to RT slowly. Residual mass persisting after RT does not indicate treatment failure. Tumor may decrease in size, but rarely disappears. Disease control is defined as the absence of progression of symptoms without any increase in size with physical examination or radiological control [18]. Also, PET scan can be used for evaluation of the metabolic response after treatment. Argiris et al. demonstrated good metabolic response with PET scan in patient with metastatic paraganglioma treated with chemotherapy [8], but RT response with PET was not presented previously. We firstly demonstrated a good metabolic response with FDG PET-CT after 2 years of RT.

## Conclusion

Glomus tumors are slow-growing lesions; therefore, it is necessary to be cautious about tumor control without increasing morbidity and mortality. Radiotherapy has become a primary treatment modality for GT in patients with unresectable symptomatic tumors, and bilateral tumors, in which, a good local control and reasonable tumor shrinkage was achieved in our case. Also we propose that FDG PET seems to be a promosing procedure for diagnosis and treatment response of GTs.

## Abbreviations

FDG: 18F-2-deoxy-D-glucose
GT: glomus tumors
MRA: magnetic resonance angiography
PET: positron emission tomography
RTOG: radiation therapy oncology group
VMA: vanilmandelic acid
